# Circular RNA circCHFR Facilitates the Proliferation and Migration of Vascular Smooth Muscle via miR-370/FOXO1/Cyclin D1 Pathway

**DOI:** 10.1016/j.omtn.2019.02.028

**Published:** 2019-04-06

**Authors:** Lei Yang, Fan Yang, Haikang Zhao, Maode Wang, Yuelin Zhang

**Affiliations:** 1 Xi’an Jiao Tong University, Xi’an City, Shanxi Province 710061, P.R. China; 2 Second Affiliated Hospital of Xi’an Medical College, Xi’an City, Shanxi Province 710038, P.R. China; 3 Department of Neurosurgery, Second Affiliated Hospital of Xi’an Medical College, Xi’an City, Shanxi Province 710038, P.R. China; 4 Department of Neurosurgery, First Affiliated Hospital of Xi’an Jiao Tong University, Xi’an City, Shanxi Province 710061, P.R. China; 5 Department of Neurosurgery, Xi’an Medical College, Xi’an City, Shanxi Province 710021, P.R. China

**Keywords:** atherosclerosis, circular RNA, circCHFR, vascular smooth muscle cells, FOXO1, CCND1

## Abstract

Circular RNA (circRNA) is a novel subgroup of noncoding RNA in the human transcriptome playing a vital role in the atherosclerosis of cerebrovascular disease. However, the in-depth mechanism by which circRNA regulates the vascular smooth muscle proliferation and migration is still elusive. Here, a novel identified circRNA, circCHFR, was validated to be aberrantly overexpressed in the ox-LDL-induced vascular smooth muscle cell (VSMCs). Functionally, the circCHFR silencing by oligonucleotide transfection suppressed the proliferation and migration ability of VSMCs. Mechanically, bioinformatics tools and luciferase reporter assay state that circCHFR acts as a sponge of miR-370, and miR-370 targets the 3′ UTR of FOXO1. Furthermore, the transcription factor FOXO1 could bind with the promoter region of CCND1 mRNA and promote Cyclin D1 expression. In summary, this finding states the vital role of the circCHFR/miR-370/FOXO1/Cyclin D1 axis and provides a profound understanding about the circRNA in smooth muscle cells and atherosclerosis.

## Introduction

Atherosclerosis (AS) is one group of the most common vascular diseases and the main cause of cerebral infarction, stroke, and cerebral ischemia-reperfusion injury, characterized by lipid metabolic disorders and originated from the intima.^1^, ^2^, ^3^, ^4^ In the pathogenesis of AS, the proliferation of vascular smooth muscle cells (VSMCs) is the typical high-probability event accompanied by the generation of collagen fibers and the accumulation of lipids.^5^ During the vasculopathy, the aberrant proliferation and migration of VSMCs from tunica media into the subendothelial layer triggers the vascular remodeling.^6^ Besides, VSMCs are exposed to complex and diverse microenvironments, in return leading to the mutability of VSMCs. Therefore, the unknown molecular mechanisms by which VSMCs participate in AS is critical for the effective treatment.

Circular RNA (circRNA) is a subgroup of noncoding RNA, characterized by the covalently closed loop generated from backsplicing.^7^, ^8^ Its molecular structure is different from that of linear RNA and single-stranded transcripts.^9^ Intrinsically, circRNA is derived from exons, introns, or intergenic regions, and its covalent loop could resist the RNase in turn to maintain its tissue specificity and abundance.^10^ Sun et al.^11^ reported that circRNA circACTA2 acts as a sponge binding miR-548f-5p and then targets 3′ UTR of α-SMA mRNA, thus upregulating α-SMA levels. Chen et al.^12^ reported that circRNA circWDR77 silencing significantly inhibited the proliferation and migration; moreover, miR-124 and fibroblast growth factor 2 (FGF-2) were downstream targets of circWDR77, forming the circWDR77-miR-124-FGF2 regulation. Therefore, the vital role of circRNA in the vascular endothelial cells is increasingly important.

In AS, circRNAs have been identified to regulate the ox-LDL-induced endothelial cells. For instance, hsa_circ_0003575 is upregulated in ox-LDL-induced HUVECs and exerts functions via potential circRNA-miRNA-mRNA network.^13^ Up to now, the relevance of circRNA with the abnormal lipid-metabolism-induced VSMC is still elusive. Herein, we performed the circRNA microarray analysis to discover the aberrantly expressed circRNA in the ox-LDL induced VSMCs. A novel identified circRNA, circCHFR (hsa_circ_0029589; gene symbol CHFR), is found to be differently upregulated in the VSMCs. Further functional experiments reveal its vital regulation on the proliferation and migration of VSMCs.

## Results

### Microarray Analysis Unveils the Aberrantly Expressed circRNA in the ox-LDL-Induced VSMCs

**Figure 1:**
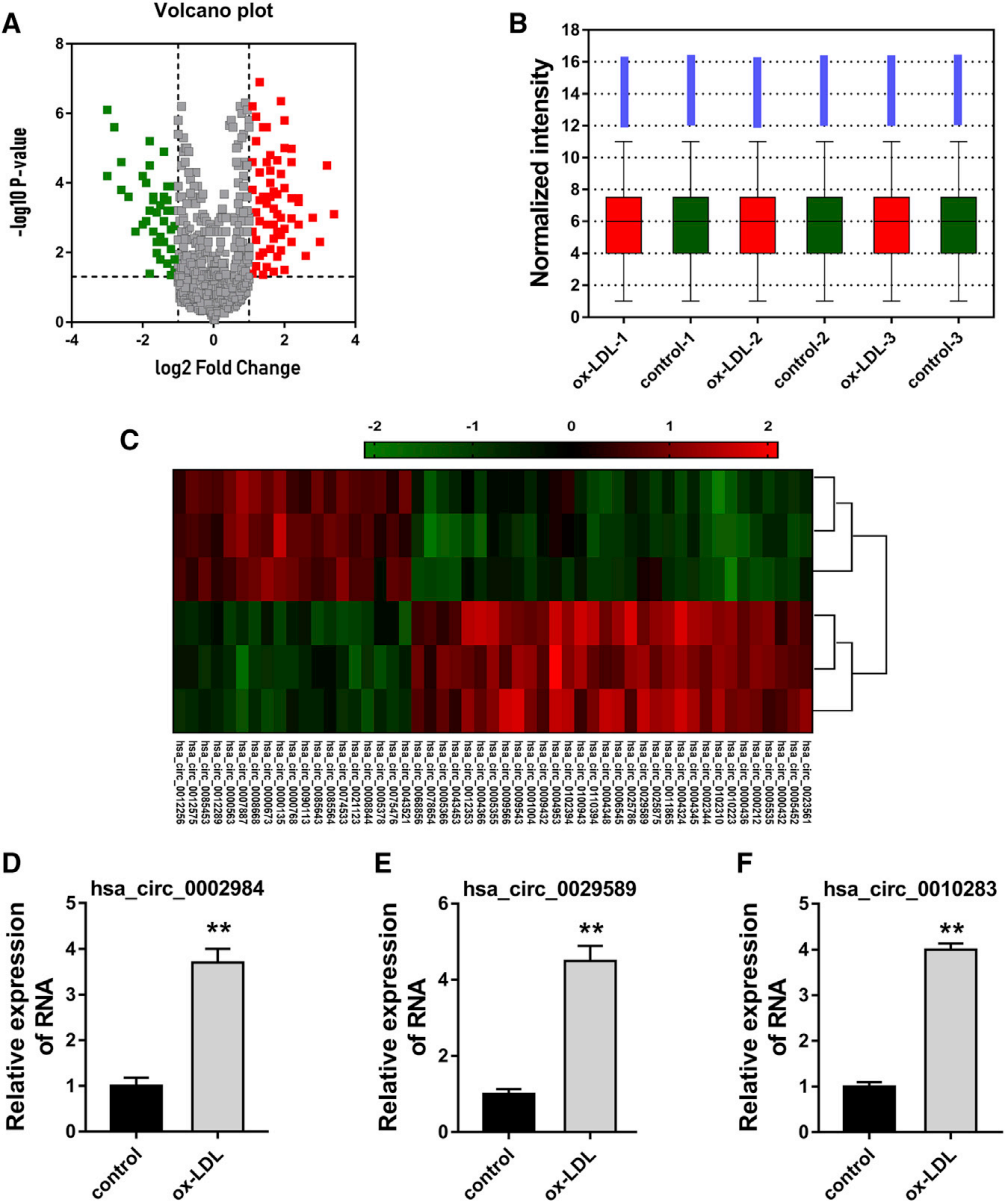
Microarray Analysis Unveils the Aberrantly Expressed circRNA in the ox-LDL-Induced VSMCs (A) Volcano plot presents the differently expressed circular RNAs (fold change > 2, p value < 0.05) in these VSMCs treated with ox-LDL and blank control. (B) The normalization of differently expressed circular RNAs. (C) Heatmap analysis visualized these circular RNA in the VSMCs. (D, E, and F) RT-PCR was performed to measure the expression of hsa_circ_0002984 (D), hsa_circ_0029589 (E), and hsa_circ_0010283 (F). *p < 0.05, **p < 0.01.

The ox-LDL was administrated to the VSMCs *in vitro* to simulate the abnormal lipid metabolism in the AS. Subsequently, microarray analysis was performed to detect the differently expressed circRNAs. Volcano plot showed the highly or lowly expressed (fold change > 2, p < 0.05) circRNAs in the data (Figure 1A). After the normalization, all the microarray data in these groups were presented in the heatmap (Figures 1B and 1C). Among these high-expressed circRNAs, we choose three for further confirmation using RT-PCR, including hsa_circ_0002984, hsa_circ_0029589, and hsa_circ_0010283 (Figures 1D–1F). These microarray analysis data unveil the aberrantly expressed circRNAs in the ox-LDL-induced VSMCs, providing an effective research strategy for circRNA.

### The Knockdown of circCHFR Suppresses the Proliferation and Migration of VSMCs

**Figure 2:**
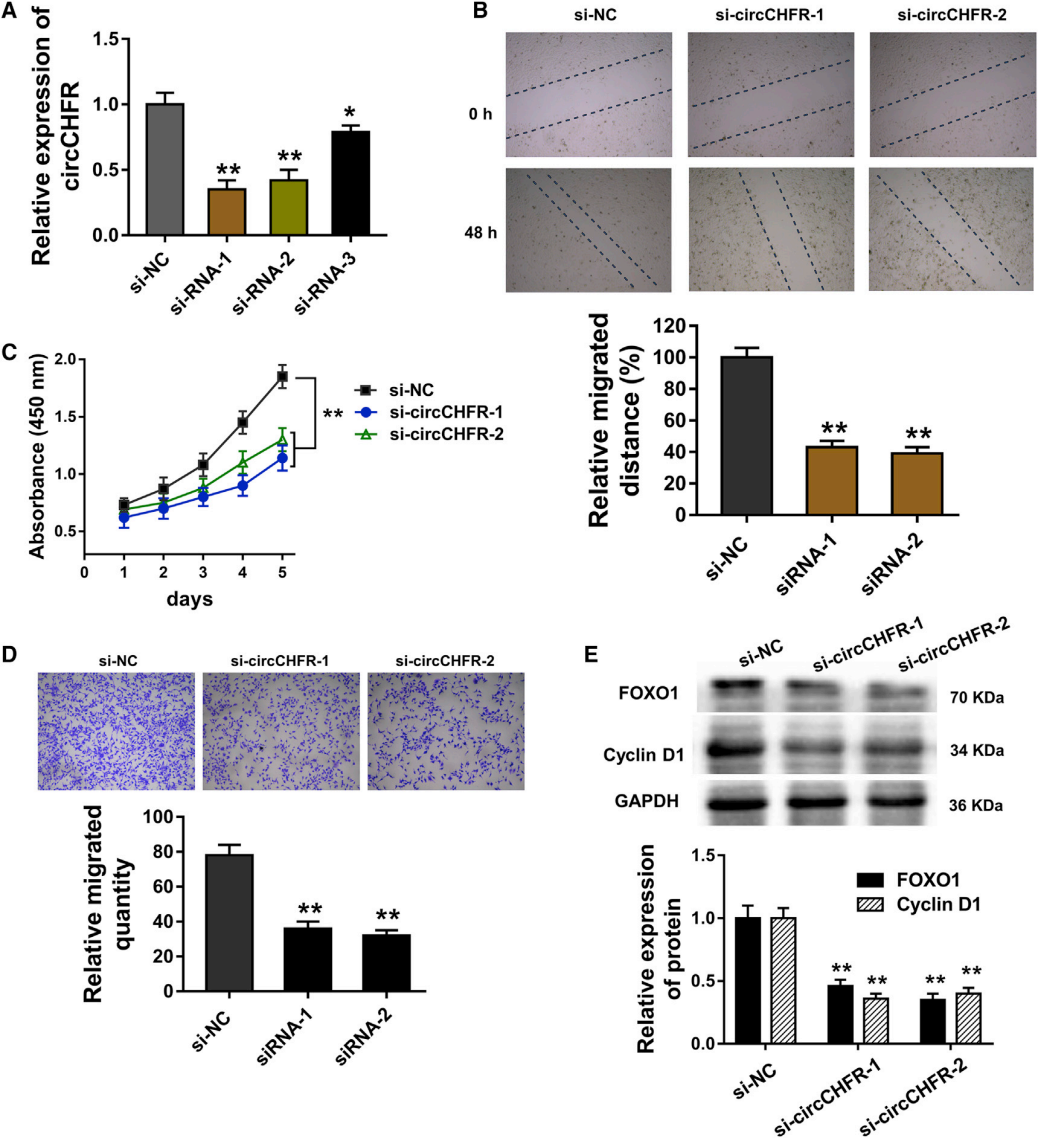
The Knockdown of circCHFR Suppresses the Proliferation and Migration of VSMCs (A) Three siRNA specifically targeting the circCHFR (si-circCHFR) were transfected into VSMCs to knock down circCHFR level. (B) Wound-healing assay for migration was performed using VSMCs transfected with two siRNAs targeting circCHFR. (C) CCK-8 assay stated the proliferative ability of VSMCs after circCHFR silencing compared with the blank transfection. (D) Transwell assay for the migration revealed the migrated VSMCs through the chamber membrane after circCHFR silencing. (E) Western blot analysis revealed the FOXO1 and Cyclin D1 protein in VSMCs with the circCHFR silencing transfection. *p < 0.05, **p < 0.01.

Previous research has revealed the candidate circRNAs in the filtration. The hsa_circ_0029589 was located in the chromosome 12, and its gene symbol is CHFR. Thus, we named it circRNA CHFR (circCHFR) in our research. Loss-of-function experiments performed using the small interfering RNA (siRNA) specifically targeting the circCHFR (si-circCHFR) were transfected into VSMCs (Figure 2A). Wound-healing assay for migration was performed using two siRNA targeting circCHFR, presenting the initiation of circCHFR silencing for the VSMCs’ migration (Figure 2B). CCK-8 assay stated that circCHFR silencing suppressed the proliferative ability of VSMCs compared with blank transfection (Figure 2C). Transwell assay for migration revealed that circCHFR silencing reduced the migrated VSMCs through the chamber membrane (Figure 2D). Western blot analysis revealed that the Forkhead box protein O1 (FOXO1) and Cyclin D1 protein were both decreased in the circCHFR-silencing transfection, acting as the downstream protein of circCHFR (Figure 2E). Therefore, these data illustrated that knockdown of circCHFR suppresses the proliferation and migration of VSMCs.

### circCHFR Functions as the Sponge of miR-370

**Figure 3:**
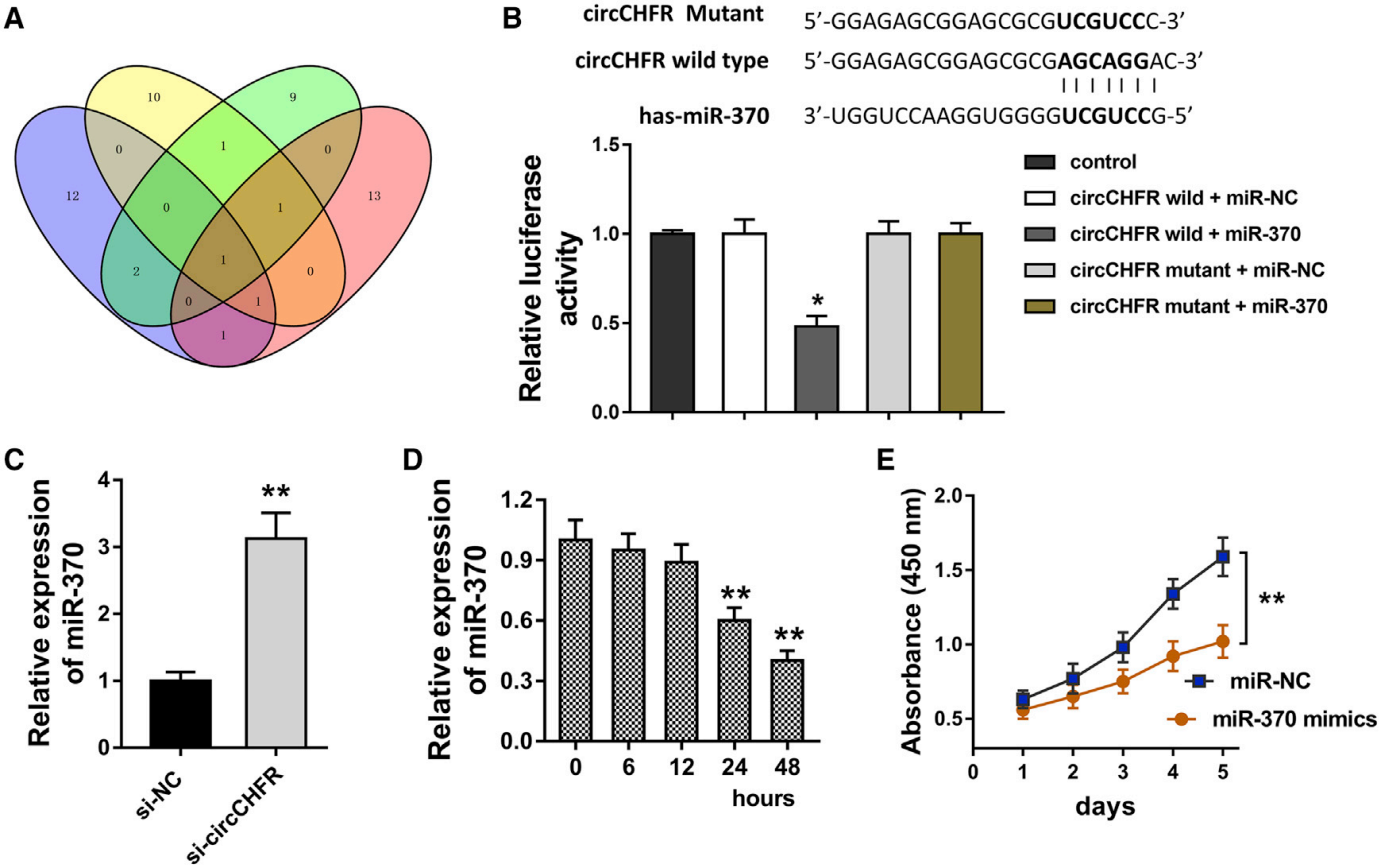
circCHFR Functions as the Sponge of miR-370 (A) Venn plot showed the potential miRNAs for circCHFR using multiple bioinformatics tools. (B) The analysis of circCHFR and miR-370 sequence indicated the several complementary binding sites within circCHFR and miR-370. Luciferase reporter assay confirmed the binding within circCHFR and miR-370. (C) RT-PCR illustrated the miR-370 level in the circRNA siRNA transfection of the VSMCs. (D) RT-PCR illustrated the miR-370 level in the VSMC-treated ox-LDL. (E) CCK-8 assay showed the proliferation of VSCs transfected with miR-370 mimics. *p < 0.05, **p < 0.01.

By now, the mechanical molecular manner for circRNA is the microRNA (miRNA) “sponge.”^14^ Therefore, we further searched the potential miRNA for circCHFR. Multiple bioinformatics tools collectively pointed out that miR-370 might act as the target for circCHFR (Figure 3A). The analysis of the circCHFR and miR-370 sequence indicated that there were several complementary binding sites within circCHFR and miR-370; moreover, a luciferase reporter assay confirmed that circCHFR could bind with miR-370 at a molecular level (Figure 3B). In VSMCs, miR-370 level was enhanced in the circRNA siRNA transfection (Figure 3C). Besides, miR-370 level was reduced in the ox-LDL-treated VSMCs (Figure 3D). CCK-8 assay found that miR-370 mimics suppressed the proliferation of VSCs (Figure 3E). These data suggest that circCHFR functions as the sponge of miR-370.

### FOXO1 Acts as the Functional Target of miR-370 in VSMCs

**Figure 4:**
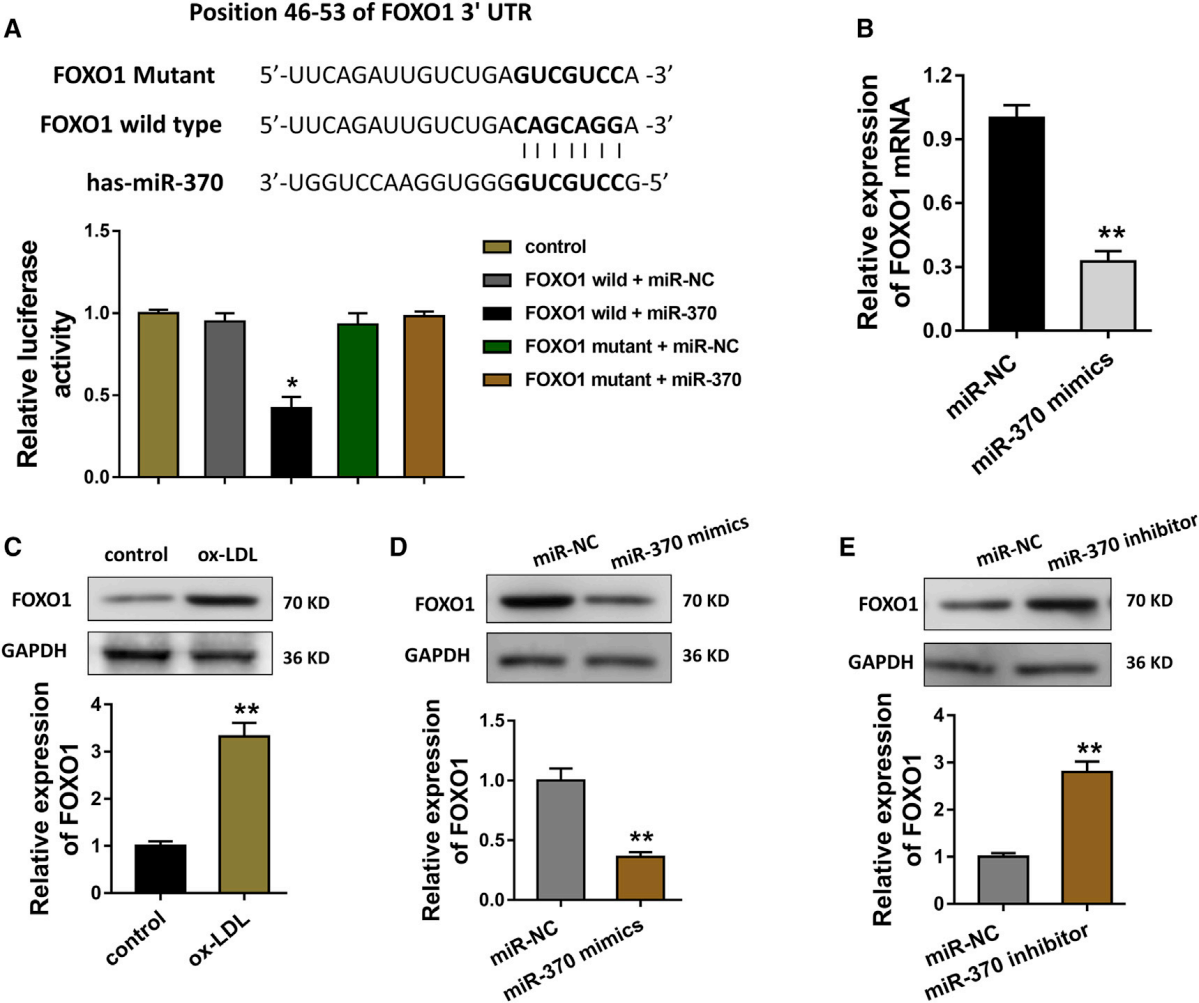
FOXO1 Acts as the Functional Target of miR-370 in VSMCs (A) FOXO1 protein functioned as the downstream target of miR-370 with complementary binding sites at the 3′ UTR. Luciferase reporter assay validated the relative fluorescence intensity when the FOXO1 3′ UTR wild-type or mutant was co-transfected with miR-370 mimics or control. (B) The transfection of miR-370 mimics could decrease the FOXO1 mRNA level in VSMCs. (C) Western blot showed the FOXO1 protein level in the ox-LDL-induced VSMCs. (D and E) The transfection of miR-370 mimics (D) or inhibitor (E) could decrease or enhance, respectively, the FOXO1 protein levels. *p < 0.05, **p < 0.01.

The results have confirmed the miRNA sponge existence of circCHFR. Next, we investigated the further role and downstream targets of miR-370 in the VSMCs. Then, the functional protein of miR-370 was predicted using the online bioinformatics tools, including miRBase (http://www.mirbase.org), miRDB (http://www.mirdb.org/), and TargetScan (http://www.targetscan.org/). Data revealed that the FOXO1 protein functioned as the downstream target of miR-370 with complementary binding sites at the 3′ UTR (Figure 4A). A luciferase reporter assay validated that the relative fluorescence intensity was markedly decreased when the FOXO1 3′ UTR wild-type was co-transfected with miR-370 mimics, demonstrating that relationship within FOXO1 and miR-370. In VSMCs, the transfection of miR-370 mimics could decrease the FOXO1 mRNA level (Figure 4B). In the ox-LDL-induced VSMCs, the FOXO1 protein level was increased compared to the control administration (Figure 4C). Moreover, the transfection of miR-370 mimics or inhibitor could decrease or enhance the FOXO1 protein levels (Figures 4D and 4E). Therefore, we confirm that FOXO1 acts as the functional target of miR-370 in VSMCs.

### FOXO1 Enhances the Transcriptional Expression of CCND1

**Figure 5:**
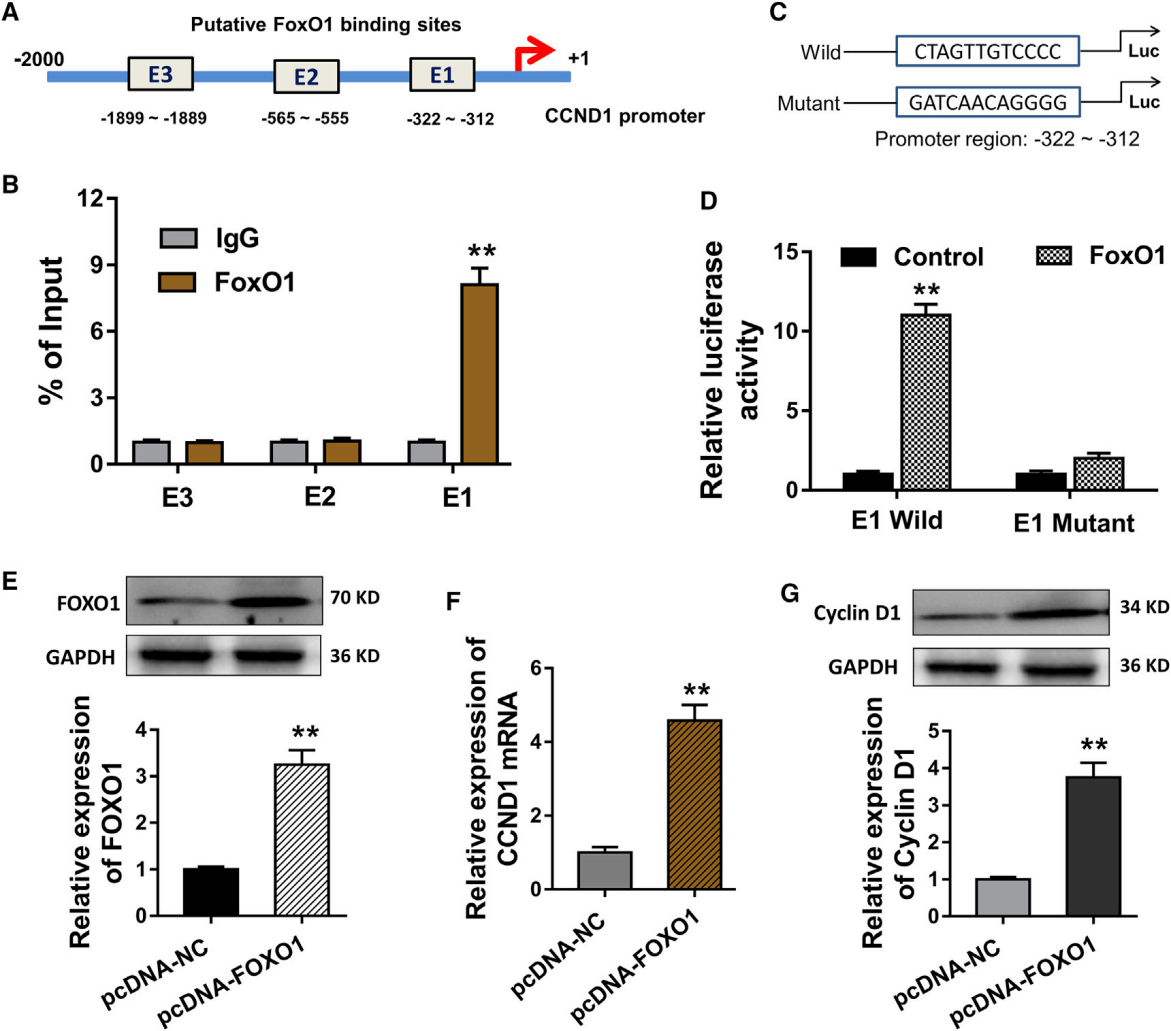
FOXO1 Enhances the Transcriptional Expression of CCND1 (A) The three putative binding elements that FOXO1 could target with the promoter region of CCND1 gene. (B) Chromatin immunoprecipitation (ChIP) assay illustrated the combining degree of FOXO1 with these element regions. (C) The wild-type and mutant element region sequence were constructed for the luciferase reporter assay. (D) Luciferase reporter assay revealed the molecular incorporation of FOXO1 and the candidate element region. (E) Western blot showed the FOXO1 level in VSMCs transfected with FOXO1 enhanced plasmid. (F) RT-PCR revealed the CCND1 mRNA. (G) Western blot showed the Cyclin D1 level in VSMCs. *p < 0.05, **p < 0.01.

**Figure 6:**
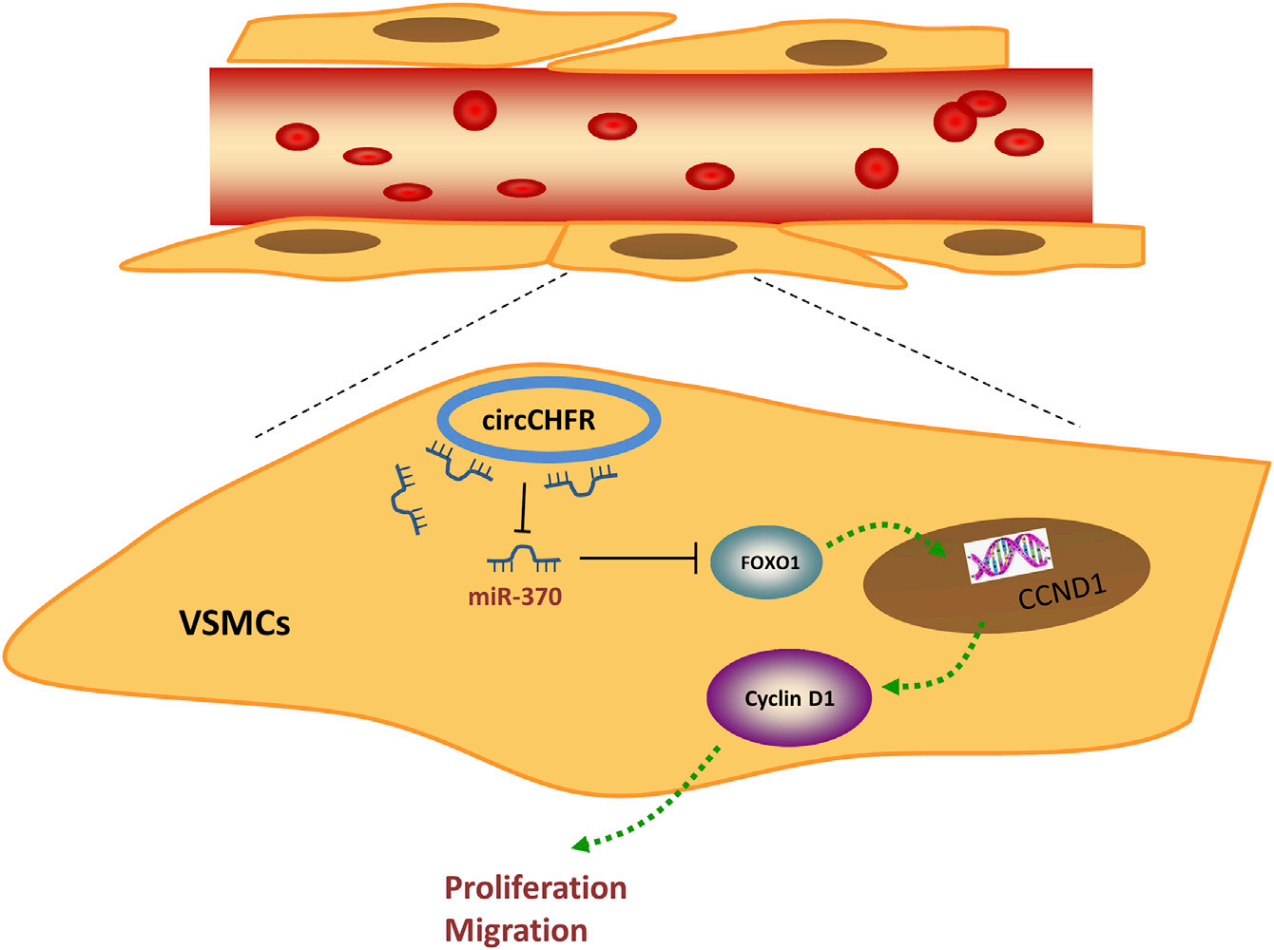
Schematic Diagram Illustrated the Role of circCHFR on the VSMCs’ Proliferation and Migration through the miR-370/FOXO1/Cyclin D1 Pathway

FOXO1 not only acted as the target of miR-370 in the present study, but also functioned as the transcription factor in the physiological and pathogenesis of human disease. In present experiments, we found that FOXO1 could target the promoter region of CCND1 gene, including three putative binding elements (Figure 5A). Then, chromatin immunoprecipitation (ChIP) assay illustrated that FOXO1 could bind with the element region (−322∼−312) (Figure 5B). After that, the wild-type and mutant element region sequence were constructed for the luciferase reporter assay, revealing the molecular incorporation of FOXO1 and the element region (Figures 5C and 5D). The FOXO1-enhanced plasmid was constructed to increase the FOXO1 level (Figure 5E) and then also improved the CCND1 mRNA (Figure 5F) expression and the Cyclin D1 level in VSMCs (Figure 5G). Thus, the data support that FOXO1 enhances the transcriptional expression of CCND1, forming the functional pathway for circCHFR on VSMCs via miR-370/FOXO1/Cyclin D1 axis (Figure 6).

## Discussion

The AS caused by multiple vasculopathy triggers severe cardiovascular disease, including coronary heart disease, cerebral infarction, and peripheral vascular disease. The proliferation and migration of vascular smooth muscle function as the significant symbols for the AS. In this study, we focus attention on the role of circRNA in the vascular smooth muscle involved in AS.

circRNAs are a vital subgroup of noncoding RNA in the human transcript, which is different from other noncoding RNA such as long coding RNA, miRNA, or piRNA.^15^, ^16^, ^17^, ^18^ The role of circRNAs in the cardiovascular disease and cardiac pathologies has been gradually recognized, including ischemia reperfusion injury, myocardial infarction, cardiac fibrosis, cardiac senescence, cardiac hypertrophy and heart failure, cardiomyopathy, AS, aneurysm, and coronary artery disease.^19^, ^20^ For instance, a novel identified circRNA, circRNA_010567, regulates the fibrosis-associated protein resection in myocardial fibrosis diabetic db/db mice, demonstrating the circRNA_010567/miR-141/transforming growth factor β (TGF-β)1 axis in the diabetic mouse myocardial fibrosis model.^21^ For other samples, references find that circDiaph3 knockdown increases the level of diaphanous-related formin-3 and promoted the differentiation of VSMCs to contractile type, and circDiaph3 promotes the Igf1r transcription and the proliferation and migration of VSMCs.^22^

In present research, we performed microarray analysis in the VSMCs to investigate the differently expressed circRNAs and finally identified the overexpressed circCHFR (hsa_circ_0029589; gene symbol CHFR).^23^, ^24^ The cellular functional experiments revealed that the knocking down of circCHFR could effectively inhibit the proliferation and migration of VSMCs *in vitro*. Therefore, the data stated that the circCHFR participates in the regulation of VSMCs’ phenotypic change. Moreover, circCHFR is also validated to be a promoter for the AS.

Regarding the molecular mechanism of circRNA in the human pathogenesis, the most canonical manner for circRNA is the miRNA sponge, involved in the human cancer, diabetic nephropathy.^25^, ^26^, ^27^ Thus, we performed bioinformatics-predictive tools to discover the potential miRNAs for the circCHFR. Fortunately, we found that miR-370 could target with the circCHFR; in return, circCHFR acts as the sponge of miR-370. More than the miR-370 sponge, the subsequent research finds that FOXO1 acts as the downstream protein of miR-370, validated by luciferase report assay. The vital pathway of circCHFR/miR-370/FOXO1 in the VSMCs has since been confirmed.

FOXO1 is also known as forkhead within the rhabdomyosarcoma, as well as a transcription factor playing an important role in regulation of gluconeogenesis.^28^, ^29^ In VSMCs, FOXO1 has been powerfully proven to regulate vascular remodeling and diabetic cardiomyopathy.^30^, ^31^ In addition, FOXO1 functions a transcription factor to modulate the human cancers.^32^ In the present research, transcription factor FOXO1 could bind with the promoter region of CCND1 to promote its transcriptional level. Thus, more evidence revealed that circCHFR regulates VSMC cellular progression via the miR-370/FOXO1/CCND1 axis. The translation product of CCND1 is the Cyclin D1 protein. In this result, the Cyclin D1 protein level is increased in the ox-LDL-administrated VSMCs and reduced when the circCHFR silenced, which is positively correlated with circCHFR and the proliferation of VSMCs.

In summary, the present study illustrates the overexpression of circCHFR in VSMCs, and its aberrant level promotes the migration and proliferation. In the mechanical level, the interaction of circCHFR, miR-370, FOXO1, and Cyclin D1 was elucidated in detail. In conclusion, this finding provides a profound understanding about the circRNA in cardiovascular diseases.

## Materials and Methods

### VSMC Culture

Human VSMCs were provided from the ATCC (American Type Culture Collection, Manassas, VA, USA). The VSMCs were cultured in a humidified incubator with 5% CO_2_ at 37°C using 10% fetal bovine DMEM supplemented with 1% 100 U/mL penicillin and 1% 100 mg/mL streptomycin. The cells were divided into two groups. One acted as the control, and the other was administrated with ox-LDL (100 μg/mL) to simulate an abnormal lipid environment. All the ethical considerations and experimental programs have been approved by the Ethics Committee of the Second Affiliated Hospital of Xi’an Medical College.

### Microarray and Computational Analysis

Microarray procedures, including RNA extraction and microarray hybridization, and data analysis were performed by Kangchen (Shanghai, China) using the Human circRNA microarray (8 × 15 K, Arraystar, Rockville, MD, USA). After the slides were washed, these acquired array images were analyzed using Agilent Feature Extraction software (version 11) and scanned by the Agilent Scanner G2505C.

### RNA Extraction and qRT-PCR

Total RNA extracted from VSMCs using Trizol reagent (Thermo Fisher Scientific, Rockford, IL, USA) was carried out initially according to manufacturer’s instructions and stored at −80°C for further assays. cDNA was reverse-transcribed and then the qRT-PCR was operated using SYBR Premix Ex Taq (Takara, Japan) on 7900 HT Real-Time PCR System (Applied Biosystems, Foster City, CA, USA). Glyceraldehyde-3-phosphate dehydrogenase (GAPDH) acts as the endogenous control. All primers in present work are listed in Table S1.

### Oligonucleotide Transfection

siRNAs targeting the back-splicing junction of circCHFR and other siRNAs were designed and synthesized by Gene Pharma Company (Shanghai, China). These siRNAs (50 nM) were transfected respectively into VSMCs (3 × 10^5^/well) with Lipofectamine 2000 (Thermo Fisher Scientific, Waltham, MA, USA). The siRNAs and negative control sequences were presented in Table S1. After 48 h, the knocking down efficiency was measured with qRT-PCR.

### CCK-8 Analysis

For the cell viability assay, the CCK-8 assay kit (Dojindo, Japan) was administrated. After transfection, VSMCs (5,000 cells/well) in 200 μL were plated in a 96-well microplate (Corning, NY, USA). The cell viability was detected at 450 nm every 24 h according to the manufacturer’s instructions.

### Wound-Healing Assay

A wound-healing assay was performed for the migration of VSMCs. VSMCs at the 95% confluence were plated on the culture dish with monolayer. A sterile 200-mL pipette tip was used to scratch across the monolayer of cells. After 24 h, cells were photographed. The distance of cell migration was measured using Image-ProPlus 4.5.1 software (Media Cybernetics, Rockville, MD, USA).

### Western Blot Analysis

The VSMCs were harvested after the oligonucleotides transfection and lysed using reagent RIPA buffer with a protease inhibitor (Roche, Basel, Switzerland). Total protein concentration was identified, and equal amounts were separated using SDS-PAGE (10%). The protein was transferred to polyvinylidene fluoride (PVDF) member (Sigma-Aldrich, St. Louis, MO, USA) and blocked in 5% non-fat milk for the following incubation with antibodies, including anti-FOXO1 and anti-Cyclin D1 (1:1000, Abcam). Finally, all these immunoreactive protein blots on the membrane were visualized with an ECL kit (Pierce, Thermo Fisher Scientific, IL, USA).

### Luciferase Reporter Assay

The plasmid (pGL3) containing the circRNA and mRNA 3′ UTR corresponding to the miR-370, as well as the CCND1 promoter region elements, were generated by PCR amplification and subcloned into the luciferase reporter vector pGL3-basic (Promega), including wild-type and mutant type. Renilla luciferase vector was also transfected in to HEK293T cells as an internal control. Luciferase activity was determined using the dual-luciferase reporter assay system and GloMax LUMINOMETER (Promega, Madison, WI, USA).

### ChIP

ChIP assay was carried out using the EZ-CHIP KIT according to the manufacturer’s instructions (Millipore, Billerica, MA, USA). FOXO1 antibody was obtained from Abcam (Hercules, CA, USA). Cross-linked chromatin was sonicated into fragments. The immunoprecipitated DNA was identified using qPCR. The data were calculated related to the input. Normal mouse immunoglobulin G (IgG) was used as a negative control.

### Statistical Analyses

All the statistical analyses involved in this research were performed using the Student’s t test for paired samples and independent samples using SPSS software. A p value less than 0.05 was considered to be of statistical significance. All data were subjected to the analyses and presented using the GraphPad Prism software.

## Author Contributions

L.Y. and H.Z. contributed to the experimental design and implementation and to the manuscript draft. F.Y., M.W., and Y.Z. performed the experiments and contributed to the data analysis.

## Conflicts of interest

All authors declare no conflicts of interest.

## Supplemental Information

Document S1. Table S1

Document S2. Article plus Supplemental Information
