# Supplementary material for: Circular RNA circCHFR Facilitates the Proliferation and Migration of Vascular Smooth Muscle via miR-370/FOXO1/Cyclin D1 Pathway
Source: Mol Ther Nucleic Acids. 2019 Apr 6;16:434–41. doi: 10.1016/j.omtn.2019.02.028 (PMC6488807; doi:10.1016/j.omtn.2019.02.028)
Supplement: Document S1. Table S1 [file mmc1.pdf]

OMTN, Volume *16*

Supplemental Information

Circular RNA circCHFR Facilitates the Proliferation and Migration of Vascular Smooth Muscle via miR-370/FOXO1/Cyclin D1 Pathway

Lei Yang, Fan Yang, Haikang Zhao, Maode Wang, and Yuelin Zhang

Supplement Table 1. Primers sequences for qRT-PCR and sequences of siRNA.

	Sequences
si-circCHFR-1	5'- CTCAGCAGTCCAGCCATACGT-3'
si-circCHFR-2	5'- AGCAGTCCAGCCATACGTCGT-3'
si-circCHFR-3	5'- TCAGCAGTCCAGCCATACGTC-3'
circCHFR	forward, 5'- CTTCCAGCCCATGCCCGACCGG-3'
	reverse, 5'- CAGAAGGCAGGCGGCGCAG-3'
miR-370	forward, 5'-GCAGCACATAATGGTTTGTG-3'
	reverse, 5'-GCAGCACATCATGGTTTACA-3'
FOXO1	forward, 5'-AGACCTACACCAAGAGTTCGCATC-3'
	reverse, 5'-CATGTGCCGTTTCATGTGCAGC-3'
CCND1	forward, 5'-CTAAGATGAAGGAGACCATCCC-3'
	reverse, 5'-AAGGTCTGCGCGTGTTTGCGGAT-3'
miR-370 inhibitor	5'-ACGGAUUCCUGGGAAAACUGGAC-3';
miR-370 mimics	5'-GTCCAGTTTTCCCAGGAATCCCT-3';
GAPDH	forward, 5'-AGAAGGCTGGGGCTCATTTG-3'
	reverse, 5'-AGGGGCCATCCACAGTCTTC-3'
